# PsychED – A National Teaching Programme to Boost Medical Student Engagement in Psychiatry

**DOI:** 10.1192/j.eurpsy.2025.921

**Published:** 2025-08-26

**Authors:** R. Hafes, D. Varma, N. Stanton, S. Thakaria

**Affiliations:** 1 Psychotherapy; 2 Liaison Psychiatry; 3 Forensic Psychiatry; 4 Children and Adolescent, CNWL, London, United Kingdom

## Abstract

**Introduction:**

In the 2019 *Choose Psychiatry* guidance for medical schools in the UK, medical students voiced a need for more extracurricular activities focused on mental health. They specifically requested the inclusion of psychiatric clinicians in their education to deepen their understanding of psychiatry and its different roles. Applications for core psychiatry training have risen dramatically, leading to oversubscription in recent years. In response to this demand, *PsychED UK*, a national teaching programme was created to further foster students’ knowledge and interest in psychiatry.

**Objectives:**

- Increase medical students’ engagement in psychiatry as a career.
- Enhance students’ knowledge and confidence in psychiatric practice.
- Boost interest in psychiatry through practical insights and exposure.

**Methods:**

Between December 2023 and February 2024, six one-hour teaching sessions were offered to medical students and Foundation Programme trainees through Medall, a virtual healthcare platform, delivered by consultants, registrars, and core trainees, ensuring a broad range of experiences.

In order to increase engagement we selected different types of presentation content: session 1 and session 4 focused on practical and personal tips on life as a psychiatry trainee, session 2 and session 3 delivered high-yield content tailored to support exam revision and lastly session 5 and session 6 gave an insight into subspecialties, providing a glimpse into exciting career pathways beyond the general practice.

Pre- and post-session questionnaires were used to measure the satisfaction scores, including likert scale ratings focusing on the student’s knowledge, confidence, and interest in a career in psychiatry and free text box for qualatative data.

**Results:**

We had a strong turnout, with 126 attendees participating across six sessions. The audience included a diverse range of individuals, from first-year medical students to Foundation Year 2 trainees, with participants joining from across the UK and even internationally. The results showed strong overall satisfaction, with all sessions scoring 77% or higher. Sessions 1, 3, and 4 received the highest satisfaction ratings, and Sessions 1 and 4 had the highest percentage of attendees providing feedback, indicating strong engagement with the content. Free-text feedback analysis highlighted that attendees were seeking more personal experiences and insights from current core trainees.

**Image 1:**

**Figure:**
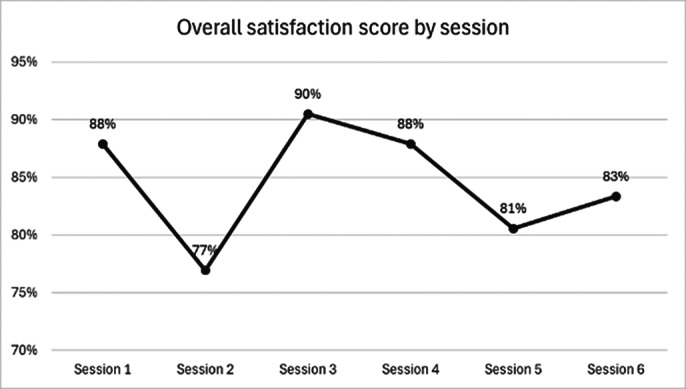

**Image 2:**

**Figure:**
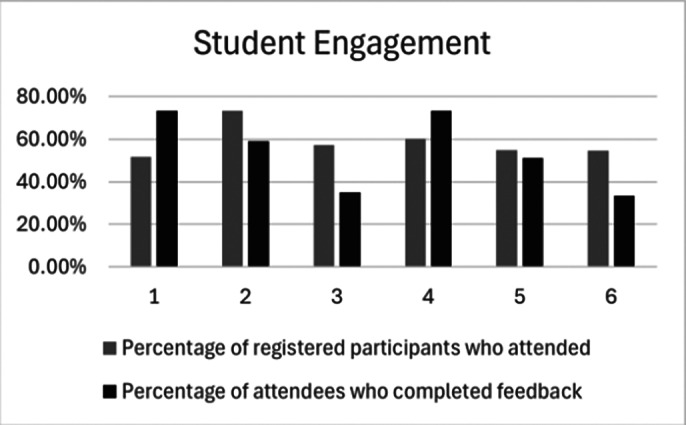

**Conclusions:**

The *PsychED UK* programme effectively enhanced medical students’ knowledge and confidence in psychiatry, addressing key areas identified in previous student feedback. Based on these positive results, a second series of teaching sessions is planned, with further refinements to the programme. Due to the high level of engagement and satisfaction with sessions 1 and 4, the second cycle will be presented by two speakers in a conversational format, focusing on the experiences of psychiatry trainees.

**Disclosure of Interest:**

None Declared

